# Snow-CLOCs: Camera-LiDAR Object Candidate Fusion for 3D Object Detection in Snowy Conditions

**DOI:** 10.3390/s24134158

**Published:** 2024-06-26

**Authors:** Xiangsuo Fan, Dachuan Xiao, Qi Li, Rui Gong

**Affiliations:** 1 School of Automation, Guangxi University of Science and Technology, Liuzhou 545006, China; 2 Guangxi Collaborative Innovation Centre for Earthmoving Machinery, Guangxi University of Science and Technology, Liuzhou 545006, China; 3 Key Laboratory of Disaster Prevention & Mitigation and Prestress Technology of Guangxi Colleges and Universities, Liuzhou 545006, China

**Keywords:** multi-modal object detection, YOLOv5, InceptionNeXt, DROR

## Abstract

Although existing 3D object-detection methods have achieved promising results on conventional datasets, it is still challenging to detect objects in data collected under adverse weather conditions. Data distortion from LiDAR and cameras in such conditions leads to poor performance of traditional single-sensor detection methods. Multi-modal data-fusion methods struggle with data distortion and low alignment accuracy, making accurate target detection difficult. To address this, we propose a multi-modal object-detection algorithm, Snow-CLOCs, specifically for snowy conditions. In image detection, we improved the YOLOv5 algorithm by integrating the InceptionNeXt network to enhance feature extraction and using the Wise-IoU algorithm to reduce dependency on high-quality data. For LiDAR point-cloud detection, we built upon the SECOND algorithm and employed the DROR filter to remove noise, enhancing detection accuracy. We combined the detection results from the camera and LiDAR into a unified detection set, represented using a sparse tensor, and extracted features through a 2D convolutional neural network to achieve object detection and localization. Snow-CLOCs achieved a detection accuracy of 86.61% for vehicle detection in snowy conditions.

## 1. Introduction

The current research hotspot lies in autonomous driving technology, where object-detection techniques serve as indispensable components. Existing object-detection methods mainly encompass approaches based on images, LiDAR, and multi-sensor fusion. However, the current research predominantly focuses on normal weather, with relatively less emphasis on the study of object-detection algorithms under adverse weather conditions.

Adverse weather conditions, such as rain, snow, and fog, pose greater challenges to the perception and decision-making capabilities of autonomous vehicles. Under adverse weather conditions, single-modal sensors are prone to environmental interference, making accurate operation difficult, thus significantly affecting the safety of vehicle travel. For example, in high or low-light scenes, the foreground and background may appear too similar, resulting in distorted image data collection, which markedly reduces the accuracy of pure visual object detection. Additionally, in snowy conditions, falling snowflakes may contaminate camera lenses, leading to image distortion, rendering pure visual-based object-detection algorithms [1,2,3,4] unable to function properly.

Currently, LiDAR is widely used in the field of autonomous driving for object detection. However, due to the sparsity and disorderliness of point clouds, pure LiDAR-based object-detection algorithms [5,6,7,8] struggle to accurately identify distant targets. Particularly in snowy conditions, the presence of snowflakes introduces significant noise into the point cloud, further affecting the performance of LiDAR algorithms. Therefore, facing these challenges, it is necessary to develop more robust object-detection algorithms to address various environmental interferences under adverse weather conditions, ensuring the stable and reliable operation of autonomous driving systems in any weather condition.

In this paper, we investigate object-detection techniques under snowy conditions and propose a solution based on the Snow-CLOCs algorithm. Our aim is to enhance object-detection technology for autonomous driving in snowy environments by employing backend fusion. The innovations of this paper are as follows:

1. To address the issue of image data distortion under adverse weather conditions, we introduce the DTCWT snow removal algorithm to process snowflakes in images and improve the quality of image data;
2. We improve upon YOLOv5 by replacing InceptionNet with the backbone network and simultaneously incorporating Wise-IoU to enhance the accuracy of image object detection;
3. To tackle the problem of point-cloud data being susceptible to snowflake interference under adverse weather conditions, we introduce the Dynamic Radius Outlier Removal (DROR) filtering algorithm to reduce point-cloud noise;
4. Finally, we propose the Snow-CLOCs post-fusion algorithm, which combines the results of YOLOv5 and SECOND object detection to enhance the accuracy of object detection.

## 2. Related Work

### 2.1. Snowy-Weather Image Processing

Snowy-weather image processing is a challenging task under extremely adverse weather conditions, which include snowflake shapes, streaks, and obscuration effects similar to foggy conditions. These environmental factors pose greater difficulties for the camera sensors of autonomous vehicles, thus requiring effective and high-quality images to support the normal operation of autonomous driving systems. Currently, algorithms for snow weather image processing can be mainly categorized into two types: traditional methods [9,10,11] and deep learning-based methods [12,13,14,15].

Traditional methods primarily rely on manually designed filtering channels, extracting color information features (such as saturation and visibility, etc.) from images to separate snowflakes and achieve snow removal effects. This method depends on the manual observation of snowflake characteristics and attempts to design filters based on these features to achieve snow removal effects. With the development of deep learning technology, deep learning-based snow removal algorithms have gradually gained attention. For example, Liu et al. [14] proposed a method using deep learning algorithms to process opaque and semi-transparent snow particles. Additionally, Chen et al. [12] proposed a snow removal method based on image obscuration effects and image restoration theory. However, existing snow weather image-processing algorithms mostly synthesize snow weather image data and typically only consider snowflake shapes while neglecting factors such as snow streaks and snowflake sizes. This limitation restricts the potential application of these algorithms in real-world scenarios, as actual snowy-weather conditions may be more complex and varied. Therefore, in practical applications, it is necessary to consider object-detection techniques further to comprehensively identify and process various features in snowy-weather images, enhancing the perception and decision-making capabilities of autonomous vehicles under snowy-weather conditions.

### 2.2. Image-Based Object-Detection Algorithms

Image object-detection algorithms can be classified into two main categories based on their detection workflow: single-stage object-detection algorithms and multi-stage object-detection algorithms. Single-stage object-detection algorithms [1,2,3] and multi-stage object-detection algorithms [4,16] each have their own advantages and disadvantages in handling image object-detection tasks.

The introduction of the YOLO algorithm marked a significant advancement in image-based object-detection technology. The latest iteration, YOLOv9 [2], incorporates groundbreaking technologies such as Programmable Gradient Information (PGI) and Generalized Efficient Layer Aggregation Network (GELAN), greatly advancing the field of real-time object detection. Fast-RCNN [4] proposed an end-to-end training method, effectively addressing the issue of multi-stage training and improving object-detection performance to a certain extent. Faster-RCNN [16] introduced a novel fully convolutional network to generate candidate regions, accelerating the selection speed of candidate boxes and further enhancing the efficiency of object detection.

For image-based object-detection technology under adverse weather conditions, most research scenarios focus on object detection in rainy and foggy weather. For instance, MS-DAYOLO [17] proposed a multi-scale domain adaptive framework. This framework utilizes multiple domain adaptive paths and corresponding domain classifiers on different scales of the YOLOv4 [18] detector to generate domain-invariant features, significantly improving the accuracy of image object detection in rainy and foggy weather. However, existing image object-detection algorithms mainly rely on the texture and semantic information of images to estimate the three-dimensional bounding boxes of objects, limited by the inability to perceive the depth of objects, resulting in lower detection accuracy. In particular, image distortion caused by snowy weather significantly impacts pure visual-based object-detection algorithms.

### 2.3. LiDAR-Based Object-Detection Algorithms

Lidar-based object-detection technology can be divided into two categories based on how point clouds are processed: algorithms based on voxelization grids [19,20,21,22,23] and those based on point feature extraction [5,6,24,25,26,27]. VoxelNet [19] proposed an end-to-end network framework based on voxelized feature extraction, which detects targets through a sliding window approach. However, the computational complexity of voxelization grids is high, especially with the increase in voxelization grid resolution, leading to geometric growth in computational costs and limiting its efficiency in practical applications. On the other hand, PointNet’s main idea is to treat point clouds as an unordered set of points and process them through neural networks to accomplish tasks such as classification and segmentation. This model can directly accept unordered point clouds as input and process the entire point cloud globally without relying on prior grid structures or manual feature extraction. However, PointNet [5] is relatively weak in extracting local features, which may result in poor performance when dealing with tasks that require local importance. In snowy environments, the accumulation of snow on the ground and objects results in sparse and incomplete point-cloud data. The reflective properties and varying shapes of snow make it difficult for LiDAR (Light Detection and Ranging) to acquire accurate and dense point-cloud data. This sparsity and incompleteness increase the difficulty of 3D object detection. Moreover, the impact on a single LiDAR sensor is particularly significant in such conditions. Snow not only degrades the quality of point-cloud data but also makes it challenging for the sensor to distinguish the contours and positions of different objects. Therefore, relying on a single LiDAR sensor for 3D object detection presents clear limitations and challenges.

### 2.4. Multi-Sensor Fusion Object-Detection Algorithms

Multi-modal fusion object-detection algorithms are a method used to process data from multiple sensors. Depending on the fusion stage, multi-modal methods can be divided into early fusion [28,29,30], deep fusion [20,31,32,33,34], and late fusion methods [35].

Early fusion [28,29,30], also known as data-level fusion, involves the fusion of raw point clouds and images. In this technique, cross-modal interaction information is effectively utilized, but it poses challenges in data alignment and has high computational requirements. Some algorithms like MVX-Net and Point Augmenting belong to early fusion algorithms.

Deep fusion [20,31,32,33,34] is a feature-level fusion method that utilizes cross-modal information from point clouds and images. It requires precise data alignment and often involves complex architectures. For example, MV3D is a two-stage algorithm that utilizes multi-view features obtained through Region of Interest (RoI) pooling for 3D object detection. Additionally, the Aggregated View Object-Detection (AVOD) network, designed for autonomous driving scenarios, combines the bird’s eye view (BEV) from LiDAR with the frontal view from cameras. MMF utilizes multi-task losses related to 2D and 3D object detection to achieve a fusion of images and point clouds. Cont Fuse employs an efficient fusion technique by transforming the camera with interpolation into the front view in BEV.

Late fusion systems [35] are relatively simple to construct because they involve pre-trained single-modal detectors and only require correlation at the detection level. For example, late fusion methods are deployed in the environmental perception modules of Baidu Apollo and the Autowar architecture at Nagoya University.

However, in snowy environments, multi-sensor fusion technology for object detection faces numerous challenges. First, snowflakes and snow accumulation affect the visual quality of camera images, causing blurriness. This not only reduces image clarity but also increases the difficulty of object detection. Additionally, in snowy conditions, the scattering and attenuation of laser signals significantly diminish the accuracy and reliability of point-cloud data. Second, due to the different responses of cameras and LiDAR to environmental conditions, the data collected by both sensors may exhibit spatial and temporal inconsistencies. Addressing these inconsistencies requires complex calibration and synchronization algorithms for data fusion, thus increasing the difficulty of multi-sensor fusion.

## 3. Approach

**Figure 1:**
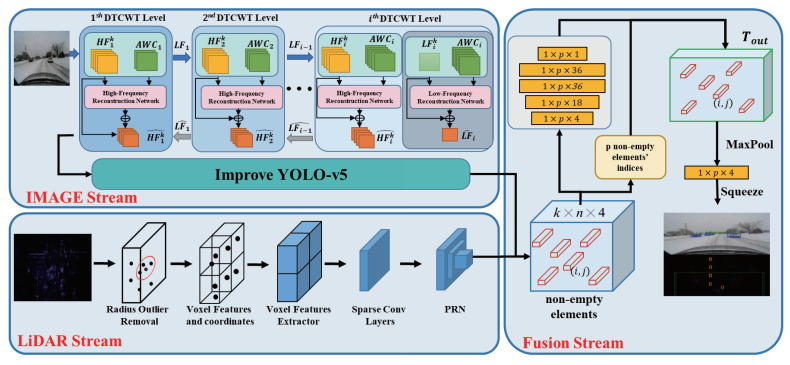
Snow-CLOCs framework, mainly consisting of the image stream, LiDAR stream, and fusion stream.

The network structure of this algorithm mainly consists of three parts: the image object-detection module, the point-cloud object-detection module, and the backend fusion module. To address the issue of image distortion caused by snowy weather, we introduce a novel hierarchical Dual-Tree Complex Wavelet Transform (DTCWT) snow removal theory [36]. By decomposing snowy-weather images into high-frequency and low-frequency components and adopting a hierarchical decomposition approach, different scales of snow can be decomposed into each sub-band, enabling precise segmentation of snowflakes of various sizes. In the image object-detection module, we enhance the detection accuracy of image targets by replacing the backbone network with InceptionNeXt [37] based on YOLOv5 and simultaneously introducing the Wise-IoU [38] algorithm. For the point-cloud object-detection part, as LiDAR point clouds in snowy-weather scenes are affected by snowflakes and may produce point-cloud noise, we utilize the Dynamic Radius Outlier Removal (DROR) filtering algorithm [39] to remove noise from the point cloud, therefore improving data quality. We then employ the SECOND [7] algorithm to detect point-cloud objects. Finally, in the backend fusion module, we utilize a backend fusion algorithm to fuse the detection results between the two sensors. We employ geometric consistency and semantic consistency to fuse our detection results, enabling the two modalities of network structures to operate independently without interference and allowing for separate training and combination. As shown in Figure 1.

### 3.1. The Snowy-Weather Image-Processing Module

In adverse environments such as snowy weather, images captured by cameras are often affected by a large number of snowflakes, snow streaks, and fogging effects, leading to degraded image quality and thus affecting the accuracy of object detection. To improve image quality and object-detection accuracy, we use Dual-Tree Complex Wavelet Transform (DTCWT) to process the images.

The Dual-Tree Complex Wavelet Transform generates complex wavelet coefficients through two parallel filter banks (tree structures). One tree uses real wavelet filters, while the other uses imaginary wavelet filters. By combining the outputs of these two trees, complex wavelet coefficients are obtained, providing both amplitude and phase information of the signal. A notable advantage of DTCWT is its good directional selectivity, enabling fine analysis of signals in multiple directions, typically resolving information at ±15 degrees, ±45 degrees, and ±75 degrees. Moreover, DTCWT retains the time-frequency localization characteristics of traditional wavelet transforms while effectively reducing spectral aliasing and artifact effects through its dual-tree structure, therefore enhancing the quality and accuracy of signal processing. The DTCWT processing workflow is illustrated in the “IMAGE Stream” section. First, at the first level, the image is decomposed into high-frequency components (HF1) and low-frequency components (LF1). Then, the low-frequency component (LF1) is further decomposed into high-frequency components (HF2) and low-frequency components (LF2), and this process continues for K levels of decomposition. In each decomposition level, we use a high-frequency reconstruction network to remove small and medium-sized snowflakes and restore the detailed information of the image. The low-frequency reconstruction network is used to remove larger snowflakes and restore the structural information of the image. Next, the low-frequency and high-frequency information is combined using the inverse DTCWT. To enhance the performance of the network, we introduce Aggregated Wavelet Components (AWC). Compared to conventional downsampling, AWC can capture more semantic and environmental information at different scales during low-frequency component reconstruction, therefore aiding in better image reconstruction.

To enhance the network’s performance, we introduced the Aggregate Wavelet Component (AWC). Unlike standard downsampling, AWC captures more diverse semantic and contextual information across different scales during low-frequency component reconstruction, therefore aiding in better image reconstruction. The design of AWC focuses on effectively capturing and integrating multi-scale semantic and contextual information to improve the quality of low-frequency component reconstruction.

First, AWC receives the low-frequency components (LF) decomposed by the DTCWT, which contain the main structural information of the image. To further capture multi-scale information, AWC employs a multi-scale feature extraction mechanism through multiple convolutional and pooling layers. Each convolutional and pooling layer has different kernel sizes and strides to extract hierarchical features ranging from local details to global structures. After extracting features at various scales, AWC integrates these features. This integration is typically accomplished through feature concatenation or feature weighting (attention mechanism). Feature concatenation directly merges the features from different scales, increasing the dimensionality of the features. Feature weighting uses attention mechanisms to weight the features based on their importance, giving higher weights to key features. The fused feature maps then undergo a series of convolutional layers to enhance the correlation and compactness of the features, further extracting and refining feature information. Finally, the feature maps generated by the AWC serve as the input for the low-frequency reconstruction network. These feature maps not only include the structural information from the original low-frequency components but also integrate multi-scale semantic and contextual information, significantly improving the quality of image reconstruction and the accuracy of object detection.

Through this multi-scale feature extraction and integration, the AWC preserves the primary structural information of the image during low-frequency reconstruction while effectively incorporating more diverse semantic and contextual information from different scales. This approach substantially enhances image reconstruction quality and object-detection accuracy.

(1) $$AWC_{i}=\left\{\begin{array}{cc} \mu \left(I\right) & i=1\\ \left[\mu \left(LF_{1}\right),\ldots \mu \left(LF_{i-1}\right),\mu \left(I\right)\right] & otherwise \end{array}\right.\;\;$$

$AWC_{i}$ and $LF_{i}$, respectively, denote the AWC and the low-frequency component at the *i*-th level, [,] represents the concatenation operation, and μ denotes the multi-pooling architecture.

**Figure 2:**
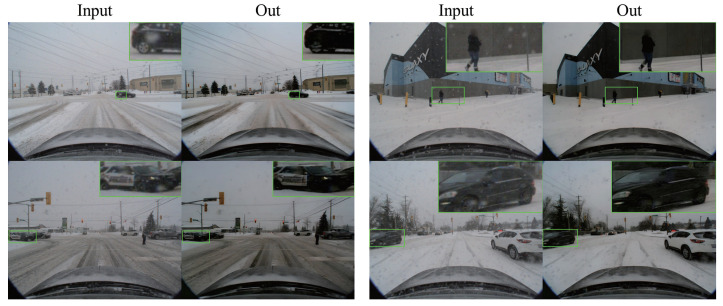
DTCWT Snowflake Removal Results. (suggested to view in enlarged mode).

In Figure 2, we present the snowflake removal performance of the DTCWT algorithm. The left panel illustrates the removal effect on larger snowflakes, while the right panel demonstrates the removal effect on smaller snowflakes. By employing the hierarchical decomposition method shown in the figure, we have effectively eliminated snowflakes from the image, resulting in significantly improved image quality.

### 3.2. Image Object-Detection Module

In snowy-weather conditions, image quality is affected by snowflakes, snow streaks, and fogging effects, which may challenge traditional YOLOv5 models. Therefore, we have made improvements by introducing the InceptionNeXt network to replace the backbone network and incorporating the Wise-IoU algorithm. The InceptionNeXt network can more effectively capture complex features in the images, thus enhancing the performance of object detection. Additionally, the Wise-IoU algorithm improves feature extraction capabilities and reduces the algorithm’s reliance on high-quality datasets, further enhancing the accuracy of object detection. Moreover, it can intelligently compute the Intersection over the Union (IoU) of object-detection boxes, therefore more accurately measuring the overlap between detection boxes, thus improving the accuracy and stability of object-detection.

**Figure 3:**
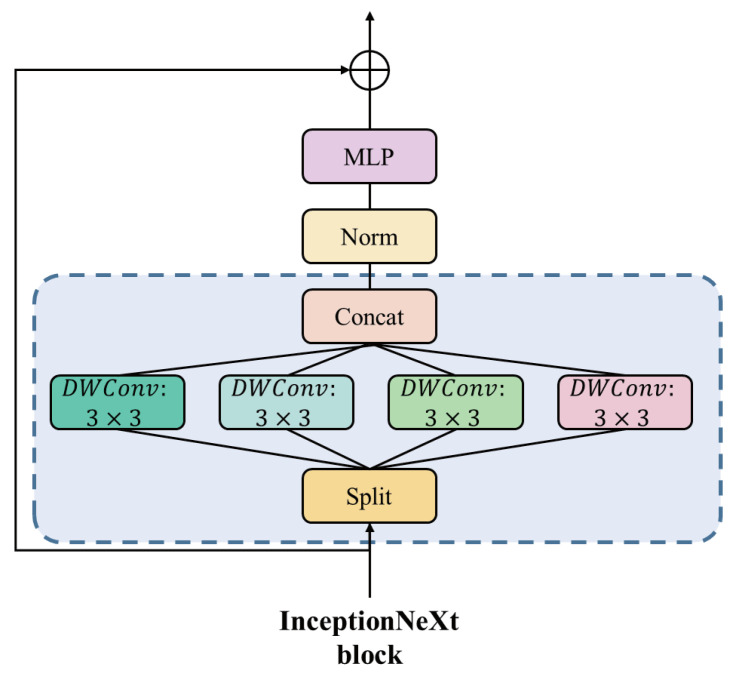
Structure of InceptionNeXt.

InceptionNeXt is an improved deep convolutional model designed to overcome the speed bottleneck caused by large kernel sizes in traditional deep convolutional models. Unlike traditional large kernel convolution operations, InceptionNeXt adopts a branching structure, with each branch using different small kernel sizes for convolution operations. This design effectively captures horizontal and vertical spatial information while avoiding the computational overhead associated with large kernels, thus improving the speed and performance of the model. The specific structure is illustrated in the Figure 3.

Specifically, we decompose the input *X* into four parts based on the channel dimension.

(2) $$\begin{array}{cc} X_{hw},X_{w},X_{h},X_{id} & =Split(X)\\ & =X_{:,:g},X_{:g:2g},X_{:2g:3g},X_{:3g:}, \end{array}$$

*g* represents the number of convolutional channels in the branch, and $X_{hw}$, $X_{w}$, $X_{h}$, $X_{id}$ are calculated as follows:

(3) $$\begin{array}{cc} X_{hw}^{\prime } & ={\mathrm{DWConv}}_{k_{s}\times k_{s}}^{g\to g}g\left(X_{hw}\right),\\ X_{w}^{\prime } & ={\mathrm{DWConv}}_{1\times k_{b}}^{g\to g}g\left(X_{w}\right),\\ X_{h}^{\prime } & ={\mathrm{DWConv}}_{k_{b}\times 1}^{g\to g}g\left(X_{h}\right),\\ X_{id}^{\prime } & =X_{id}. \end{array}$$

$k_{s}$ represents the default small kernel size, set to 3, while $k_{b}$ represents the default large kernel size, set to 11. Finally, the outputs of each branch are concatenated together.

(4) $$X^{\prime }=\mathrm{Concat}\left(X_{hw}^{\prime },X_{w}^{\prime },X_{h}^{\prime },X_{id}^{\prime }\right)$$

In snowy-weather scenes, where the quality of datasets is often low and uneven, existing bounding box regression (BBR) loss functions struggle to adapt. To address this issue, we employ Wise-IoU for data handling. Wise-IOU utilizes a dual-layer attention mechanism aimed at enhancing detection performance and accuracy. This dual-layer attention mechanism consists of two key components: distance attention function and intersection over union (IoU) adjustment. The distance attention function adjusts the attention weights of each anchor box based on the distance relationship between objects, thus aiding in improving the detection performance of distant objects. The second layer of the attention mechanism adjusts attention further by enlarging the IoU of ordinary-quality anchor boxes while reducing the IoU of high-quality anchor boxes. This adjustment helps improve the detection accuracy of ordinary targets while reducing over-detection of high-quality targets, achieving better balance. Integrating both layers of attention mechanism, Wise-IoU can more accurately capture target areas, enhancing the accuracy and robustness of object detection. The design of this dual-layer attention mechanism allows Wise-IoU to exhibit excellent performance in various object-detection tasks, particularly demonstrating significant advantages in handling distant objects. Specifically, the definition of Wise-IoU is as follows:

(5) $$L_{WIoUv3}=rR_{WIoU}L_{IoU}xc$$

(6) $$R_{WIoU}=\exp\left(\frac{{\left(x-x_{gt}\right)}^{2}+{\left(y-y_{gt}\right)}^{2}}{{\left(W_{g}^{2}+H_{g}^{2}\right)}^{*}}\right)$$

(7) $$r^{2}=\frac{\beta }{\delta \alpha^{\beta -\delta }}$$

where $W_{g}$ represents the width of the minimum bounding box, $H_{g}$ represents the height of the minimum bounding box, and $W_{g}^{2}+H_{g}^{2}$ represents the diagonal length of the minimum bounding box. * denotes a separation operation used to prevent gradients that impede convergence in Wise-IoU. Here, *C* is a constant value, and δ ensures that r=1 when β=δ.

### 3.3. LiDAR Point-Cloud Object-Detection Module

In snowy-weather conditions, data collected by LiDAR is affected by snowflakes, raindrops, etc., resulting in a significant amount of noise in the point cloud, thus affecting the accuracy of point-cloud object detection. To address this issue, we introduced a point cloud filtering algorithm based on the Second algorithm. The traditional Radius Outlier Removal Filter algorithm has some limitations in dealing with point-cloud noise. It uses the same search radius to find neighboring points and removes points with fewer neighboring points than the specified minimum number of neighbors. However, this method does not consider the sparsity of distant point clouds, resulting in the incorrect removal of many useful pieces of information. To overcome this problem, we introduced the Dynamic Radius Outlier Removal (DROR) filtering algorithm. This algorithm dynamically adjusts the search radius size for each point and adaptively identifies and removes outliers based on their local density, thus improving the accuracy and efficiency of outlier removal.

In the DROR algorithm, the search radius $SR_{i}$ is dynamically calculated based on the local density of each point *p*. First, the search radius is calculated based on the expected point spacing when the laser beam is perpendicular to the surface reflected by LiDAR and then adjusted to account for increased point spacing on surfaces not perpendicular to the laser beam. Additionally, a minimum search radius is specified to avoid using very small search radii for points close to the vehicle. If a point does not have a sufficient number of points within the specified search radius, it is classified as an outlier. This method of dynamically adjusting the search radius based on local density allows for better adaptation to point-cloud structures with different densities and distributions, thus improving efficiency and accuracy in noise filtering and outlier removal.

**Figure 4:**
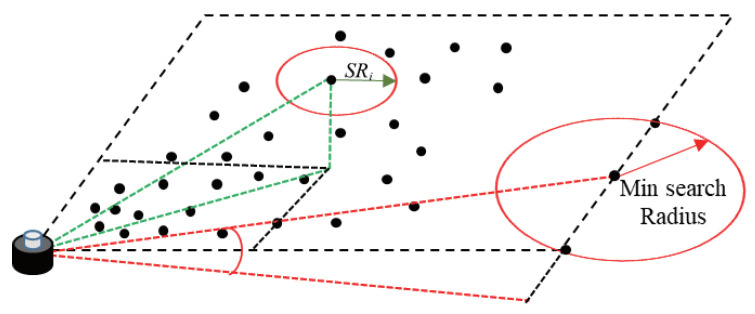
Dynamic Radius Outlier Removal (DROR) Filter Algorithm.

Figure 4 illustrates the principle of the Dynamic Radius Outlier Removal (DROR) filtering algorithm. This algorithm dynamically adjusts the search radius (Search Radius, $SR_{i}$) to better accommodate varying densities and distributions within point-cloud structures, therefore enhancing efficiency and accuracy in noise filtering and outlier removal. In the figure, the red ellipse represents the current search radius ($SR_{i}$), which is dynamically adjusted based on local density to adapt to changes in point-cloud density. Additionally, the figure shows the minimum search radius (Min Search Radius), indicated by a red arrow. This minimum search radius ensures effective searching and outlier detection even in low-density areas. The black dots in the figure represent data points in the point cloud, illustrating the density variations in different regions. The algorithm demonstrates the adjustment process of using different search radii in various density regions through green and red dashed lines.

### 3.4. Multi-Sensor Fusion-Detection Module

To improve object-detection accuracy by fusing the detection results from cameras and LiDARs in snowy-weather conditions, we adopted a backend fusion-based object-detection algorithm. This algorithm first converts the detection results from cameras and LiDARs into a consistent joint detection set represented using sparse tensors. Subsequently, we employ a 2D convolutional neural network (CNN) to process this sparse input tensor to extract relevant features. Finally, through max-pooling operations, we map the processed features to a probability score map to accomplish the tasks of object detection and localization.

In image object-detection tasks, the output of 2D object detection consists of a set of 2D detection boxes and confidence scores on the plane image. For *N* candidate boxes on an image, they can be defined as:

(8) $$\begin{array}{cc} B^{2D} & =\{B_{1}^{2D},B_{2}^{2D},\ldots ,B_{n}^{2D}\},\\ B_{k}^{2D} & =\{\left[x_{k1},y_{k1},x_{k2},y_{k2}\right],s_{k}^{2D}\} \end{array}$$

$B^{2D}$ represents a set of *n* candidate boxes on an image. For the *k*-th 2D detection candidate box in this set, $x_{k1},y_{k1}$ represents the pixel coordinates of the top-left corner of the candidate box, $x_{k2},y_{k2}$ represents the pixel coordinates of the bottom-right corner of the candidate box, and $s_{k}^{2D}$ represents the confidence score.

In LiDAR point-cloud object-detection tasks, the output of 3D object detection consists of the dimensions (height, width, length) of the candidate box, the position (*x*, *y*, *z*) of the 3D object, and the rotation angle θ of the 3D candidate box. For a template detection result of a LiDAR point cloud, it can be defined as:

(9) $$\begin{array}{cc} B^{3D} & =\{B_{1}^{3D},B_{2}^{3D},\ldots ,B_{n}^{3D}\},\\ B_{k}^{3D} & =\{\left[h_{k},w_{k},l_{k},x_{k},y_{k},z_{k},\theta_{k}\right],s_{k}^{3D}\} \end{array}$$

$B^{3D}$ represents a set of *n* 3D candidate boxes on a point cloud. For the *k*-th 3D detection candidate box $B_{k}^{3D}$, $\left[h_{k},w_{k},l_{k},x_{k},y_{k},z_{k},\theta_{k}\right]$ represents the 7 parameters of the point-cloud candidate box. $s_{k}^{3D}$ represents the confidence score of the point-cloud candidate box.

As discussed earlier, due to the limitations of a single sensor, some correct candidate boxes may be suppressed. To address this, we adopt a new fusion network architecture to reevaluate all candidate boxes by constructing a tensor *T* representing k×n×4.

(10) $$T_{i,j}=\left\{IoU_{i,j},s_{i}^{2D},s_{j}^{3D},d_{j}\right\}$$

where $IoU_{i,j}$ is the IoU between the *i*th 2D detection and the *j*th projection of the 3D detection, $s_{i}^{2D}$ and $s_{j}^{3D}$ are the confidence scores of the *i*th 2D detection and the *j*th 3D detection, respectively. $d_{j}$ represents the normalized distance between the *j*th 3D bounding box and the LiDAR on the x–y plane. Elements in $T_{i,j}$ with an IoU of zero will be eliminated as they are geometrically inconsistent.

Our fusion network is designed to handle non-empty bounding boxes that intersect between the 3D detection boxes for each projection and the 2D detections. However, there are still many 3D detections that do not correspond to any 2D boxes. In such cases, we fill the information of the 3D detection into the last element of *T* and set $IoU_{i,j}$ and $s_{k}^{2D}$ to −1. This is because 3D detection can identify objects that 2D detection misses, and thus, we retain this information. This allows our network to distinguish between such cases and other examples with very small IoU and $s_{k}^{2D}$.

## 4. Experiments

### 4.1. Dataset and Metric

Our multi-sensor fusion algorithm was evaluated on the CADC dataset [40]. This dataset, collected by Matthew Pitropov on the Autonomoose autonomous vehicle platform, aims to provide data specifically for adverse driving conditions. It was gathered during winter in the Waterloo region of Canada and is the first dataset tailored for adverse driving conditions for autonomous vehicles. The dataset comprises 56,000 camera images, 7000 LiDAR scans, and data from 75 scenes. Each scene consists of 50 to 100 frames, with annotations for each scene category. We selected data captured by LiDAR under snowy conditions and data captured by the front-facing camera for our object-detection tests. The original dataset was partitioned into training, validation, and test sets following a 6:2:2 ratio for algorithm evaluation and performance testing.

Our main focus is on the detection results in 3D and bird’s-eye view perspectives, providing validation results. Specifically, we followed benchmarks and previous studies for the vehicle category, reporting the average precision (AP) at IOU thresholds of 0.5 and 0.7. We use $AP_{BEV}$ and $AP_{3D}$ to refer to the average precision for bird’s-eye view and 3D detection tasks, respectively.

For the image and LiDAR object detectors, we improved the YOLOv5-7.0 version and the SECOND algorithm using PyTorch 1.5 with open hyperparameters as set in the code. The total training cycle was 300 epochs, with a batch size of 16 and an image size of 640 pixels. The optimizer used for training was SGD, with the multi-scale training option enabled.

In the CLOCs fusion part, the training cycle was 160 epochs, with a batch size of 1 and a maximum voxel number of 16,000, using 3 worker threads with point shuffling enabled. The noise parameters were set as follows: ground truth localization noise standard deviation was [1.0, 1.0, 0.5], ground truth rotation uniform noise range was [−0.78539816, 0.78539816], and global rotation and scaling uniform noise ranges were [−0.78539816, 0.78539816] and [0.95, 1.05], respectively.

### 4.2. Experiments Results

To demonstrate the performance improvement achieved by our modifications to the YOLOv5 model, we conducted a series of ablation experiments on the adverse weather dataset, evaluating each enhancement module using the same training strategy. The specific results are shown in Table 1.

First, we combined YOLOv5 with InceptionNeXt and found that InceptionNeXt exhibited certain advantages in vehicle object detection due to its efficient information-capturing capability. Compared to the regular YOLOv5 model, the combined model showed a peak mAP improvement of 0.3%. Second, we introduced the Wise-IoU mechanism, which reduced the reliance on high-quality datasets, making the model more suitable for adverse weather object detection. Results showed that the YOLOv5 model combined with WIOU achieved a peak mAP improvement of 1.1% compared to the regular YOLOv5 on the adverse weather dataset. Furthermore, by combining InceptionNeXt and WIOU, we found that the advantages of both modules were complementary and not conflicting. This indicates that using both modules simultaneously can effectively enhance detection performance. Finally, combining these two modules, our model achieved a peak mAP of 82% on the adverse weather dataset, representing a 1.6% improvement over regular YOLOv5.

**Figure 5:**
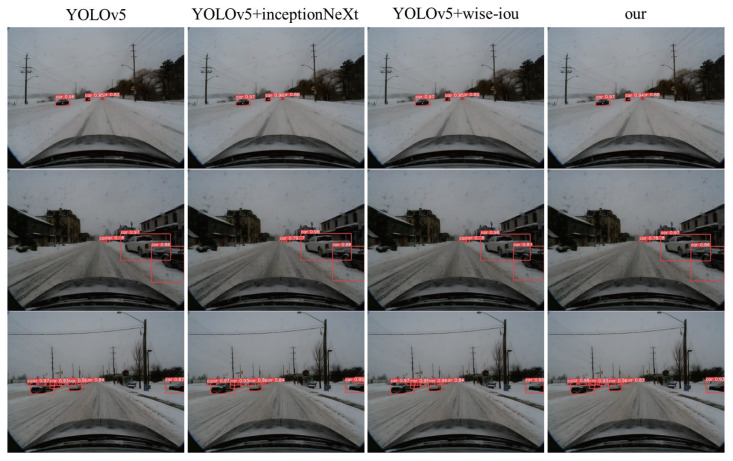
YOLO Ablation Experiment Visualization Results (suggested to view in enlarged mode).

In Figure 5. We display the visual results of YOLOv5, YOLOv5+InceptionNeXt, YOLOv5+WIOU, and YOLOv5+InceptionNeXt+WIOU. It is evident that compared to the original YOLOv5, our algorithm improves the accuracy of image object detection.

In Table 2. To demonstrate the overall performance improvement of our algorithm, we conducted ablation experiments comparing the enhanced Snow-CLOCs algorithm with the original CLOCs algorithm. Initially, we incorporated Improved YOLOv5 into the original CLOCs to improve the accuracy of 2D bounding boxes, resulting in an 8.86% increase in $A_{BEV}$ and an 18.56% increase in $A_{3D}$ in the easy mode. This enhancement addresses the poor fusion effect between 3D boxes and images due to the lower precision of the original CLOCs’ image-based object-detection algorithm. Subsequently, integrating the image noise filtering algorithm DCTW further improved $A_{BEV}$ and $A_{3D}$ by 1.99% and 3.04%, respectively, in the easy mode. Finally, by incorporating the Dynamic Radius Outlier Removal (DROR) filtering algorithm into the point-cloud part of our algorithm, we achieved an overall $A_{BEV}$ and $A_{3D}$ of 86.61% and 70.72%, respectively, in the easy mode.

To better demonstrate the performance of the Snow-CLOCs algorithm in snowing conditions, we conducted experimental tests using the Canadian Adverse Driving Conditions dataset (CADC). We compared the performance of the Snow-CLOCs algorithm with AVOD, F-PointNet, and TANet algorithms on this dataset, and the results are shown in Table 3.

As shown in Table 3, we adopted the evaluation criteria from the KITTI dataset [41,42], categorizing targets into easy, moderate, and hard cases, corresponding to targets within 30 m, targets between 30 and 100 m, and targets beyond 100 m, respectively. We evaluated their detection accuracy on the bird’s-eye view and the precision of 3D detection boxes.

Our algorithm excels in these metrics. Specifically, at an IOU of 0.7, our algorithm demonstrates outstanding performance in the easy mode. This is attributed to our algorithm’s utilization of image and point-cloud data processing techniques, greatly reducing noise interference, resulting in a bird’s-eye view detection accuracy of 86.61% and a 3D detection box detection accuracy of 70.72%. Our algorithm combines the advantages of image and LiDAR point-cloud object detection, performing excellently even in moderate mode. In this mode, our algorithm achieves a detection accuracy of 79.79% on the bird’s-eye view and 78.86% on the 3D detection boxes. Additionally, our algorithm utilizes the multi-scale object-detection capability of YOLOv5, significantly alleviating the shortcomings of LiDAR in long-distance object detection. This allows our algorithm to perform remarkably well even in the hard mode, with a bird’s-eye view detection accuracy of 78.86% and a 3D detection box detection accuracy of 62.72%.

**Figure 6:**
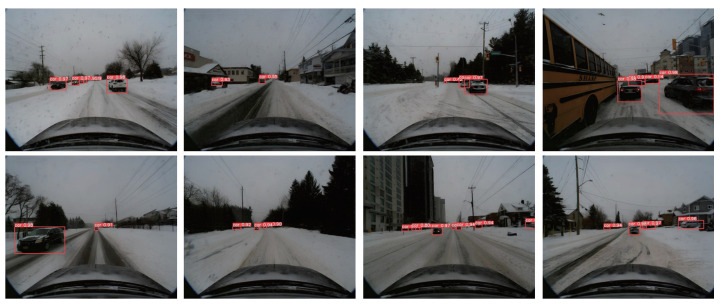
We present the 2D object-detection results of our algorithm on the validation set of the CADC public dataset. The red boxes indicate the target locations predicted by our algorithm, and the corresponding numbers represent the confidence scores (suggested to view in enlarged mode).

**Figure 7:**
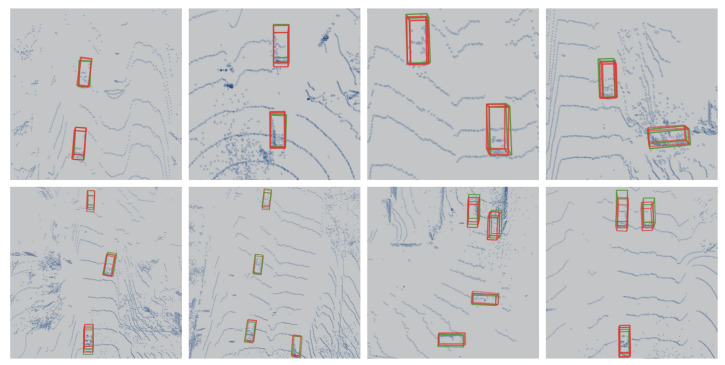
We present the 3D object-detection results of our algorithm on the validation set of the CADC public dataset. The red boxes indicate the target locations predicted by our algorithm, while the green boxes represent the ground truth target locations (suggested to view in enlarged mode).

As shown in Figure 6 and Figure 7, we present the detection results of our algorithm on the validation set. These results highlight the outstanding performance of our algorithm in snowy environments. Despite the visual challenges typically posed by snowy conditions, such as targets being covered by snow and poor ambient lighting, our algorithm consistently achieves stable object detection. This demonstrates the robustness and accuracy of our algorithm in complex environments.

## 5. Results

The contribution of this study lies in enriching the research on object-detection algorithms under adverse weather conditions and proposing an effective multi-sensor information fusion strategy. We improved the YOLOv5 object-detection algorithm by introducing the InceptionNeXt network to replace the backbone network and combining it with the Wise-IoU algorithm. Additionally, we performed backend fusion of the improved YOLOv5 algorithm with the point-cloud object-detection algorithm, achieving multi-sensor information fusion. By leveraging both image and point-cloud data, our algorithm achieved significant improvements in object-detection accuracy under adverse weather conditions such as snow. Experimental results demonstrate that our algorithm not only enhances the accuracy of object detection but also effectively addresses the problem of single-sensor susceptibility to environmental interference in adverse weather conditions.

## Figures and Tables

**Table 1:** YOLOv5 Ablation Experiments.

Model	P	R	Map50	Map50-95
YOLOv5	85.3%	71.3%	80.4%	50.9%
YOLOv5+InceptionNeXt	87.3%	70.9%	80.7%	51.5%
YOLOv5+WIOU	88.4%	71.3%	81.5%	52.1%
**our**	**87.6%**	**71.9%**	**82.0%**	**52.2%**

**Table 2:** Ablation Experiments of Snow-CLOCs Algorithm.

Model	$A_{\mathrm{BEV}}$			$A_{3D}$		
	Easy	Moderate	Hard	Easy	Moderate	Hard
CLOCs	75.59%	74.08%	73.17%	46.59%	47.32%	46.56%
CLOCs+Improved YOLOv5	84.45%	78.47%	77.72%	65.15%	59.46%	58.27%
CLOCs+DTCWT+Improved YOLOv5	86.44%	79.16%	78.17%	68.19%	60.54%	59.00%
**Our**	**86.61%**	**79.79%**	**78.86%**	**70.72%**	**63.47%**	**62.72%**

**Table 3:** Car Detection Results on the CADC Validation Set.

Model	$A_{\mathrm{BEV}}$			$A_{3D}$		
	Easy	Moderate	Hard	Easy	Moderate	Hard
AVOD	81.32%	71.86%	66.57%	68.75%	63.68%	52.54%
F-PointNet	81.40%	70.29%	62.19%	68.73%	64.53%	57.91%
F-ConvNet	80.56%	71.68%	63.07%	64.86%	57.55%	55.25%
TANet	84.67%	82.33%	80.99%	67.53%	62.12%	62.71%
**Our**	**86.61%**	**79.79%**	**78.86%**	**70.72%**	**63.47%**	**62.72%**

## Data Availability

The data used to support the results of this study are included in the article.

## References

[1] Jocher G., Chaurasia A., Stoken A., Borovec J., Kwon Y., Michael K., Fang J., Yifu Z., Wong C., Montes D. (2022). ultralytics/yolov5: v7. 0-yolov5 sota realtime instance segmentation. Zenodo.

[2] Wang C.Y., Yeh I.H., Liao H.Y.M. (2024). YOLOv9: Learning What You Want to Learn Using Programmable Gradient Information. arXiv.

[3] Wang C.Y., Bochkovskiy A., Liao H.Y.M. YOLOv7: Trainable bag-of-freebies sets new state-of-the-art for real-time object detectors. Proceedings of the IEEE/CVF Conference on Computer Vision and Pattern Recognition.

[4] Girshick R. Fast r-cnn. Proceedings of the IEEE International Conference on Computer Vision.

[5] Qi C.R., Su H., Mo K., Guibas L.J. Pointnet: Deep learning on point sets for 3d classification and segmentation. Proceedings of the IEEE Conference on Computer Vision and Pattern Recognition.

[6] Qi C.R., Yi L., Su H., Guibas L.J. (2017). Pointnet++: Deep hierarchical feature learning on point sets in a metric space. Adv. Neural Inf. Process. Syst..

[7] Yan Y., Mao Y., Li B. (2018). Second: Sparsely embedded convolutional detection. Sensors.

[8] Shi S., Wang X., Li H. PointRCNN: 3D Object Proposal Generation and Detection From Point Cloud. Proceedings of the 2019 IEEE/CVF Conference on Computer Vision and Pattern Recognition (CVPR).

[9] Pei S.-C., Tsai Y.-T., Lee C.-Y. Removing rain and snow in a single image using saturation and visibility features. Proceedings of the 2014 IEEE International Conference on Multimedia and Expo Workshops (ICMEW).

[10] Wang Y., Liu S., Chen C., Zeng B. (2017). A Hierarchical Approach for Rain or Snow Removing in A Single Color Image. IEEE Trans. Image Process..

[11] Yu S., Zhao Y., Mou Y., Wu J., Han L., Yang X., Zhao B. Content-Adaptive Rain and Snow Removal Algorithms for Single Image. Proceedings of the International Symposium on Neural Networks.

[12] Chen W.T., Fang H.Y., Ding J.J., Tsai C.C., Kuo S.Y. JSTASR: Joint Size and Transparency-Aware Snow Removal Algorithm Based on Modified Partial Convolution and Veiling Effect Removal. Proceedings of the European Conference on Computer Vision.

[13] Li Z., Zhang J., Fang Z., Huang B., Jiang X., Gao Y., Hwang J.N. (2019). Single Image Snow Removal via Composition Generative Adversarial Networks. IEEE Access.

[14] Liu Y.F., Jaw D.W., Huang S.C., Hwang J.N. (2018). DesnowNet: Context-Aware Deep Network for Snow Removal. IEEE Trans. Image Process..

[15] Zheng X., Liao Y., Guo W., Fu X., Ding X. Single-image-based rain and snow removal using multi-guided filter. Proceedings of the International Conference on Neural Information Processing.

[16] Ren S., He K., Girshick R., Sun J. (2015). Faster r-cnn: Towards real-time object detection with region proposal networks. IEEE Trans. Pattern Anal. Mach. Intell..

[17] Hnewa M., Radha H. (2021). Multiscale domain adaptive yolo for cross-domain object detection. Proceedings of the 2021 IEEE International Conference on Image Processing (ICIP).

[18] Bochkovskiy A., Wang C.Y., Liao H.Y.M. (2020). Yolov4: Optimal speed and accuracy of object detection. arXiv.

[19] Yang B., Luo W., Urtasun R. PIXOR: Real-time 3D Object Detection from Point Clouds. Proceedings of the 2018 IEEE/CVF Conference on Computer Vision and Pattern Recognition.

[20] Ku J., Mozifian M., Lee J., Harakeh A., Waslander S.L. (2018). Joint 3d proposal generation and object detection from view aggregation. Proceedings of the 2018 IEEE/RSJ International Conference on Intelligent Robots and Systems (IROS).

[21] Li B. 3D fully convolutional network for vehicle detection in point cloud. Proceedings of the IEEE International Conference on Intelligent Robots and Systems.

[22] Li X., Guivant J., Kwok N., Xu Y., Li R., Wu H. (2019). Three-dimensional backbone network for 3D object detection in traffic scenes. arXiv.

[23] Yang B., Liang M., Urtasun R. (2018). HDNET: Exploiting HD Maps for 3D Object Detection. arXiv.

[24] Lang A.H., Vora S., Caesar H., Zhou L., Yang J., Beijbom O. Pointpillars: Fast encoders for object detection from point clouds. Proceedings of the IEEE/CVF Conference on Computer Vision and Pattern Recognition.

[25] Mao J., Wang X., Li H. Interpolated convolutional networks for 3d point cloud understanding. Proceedings of the IEEE/CVF International Conference on Computer Vision.

[26] Yang Z., Sun Y., Liu S., Jia J. 3DSSD: Point-Based 3D Single Stage Object Detector. Proceedings of the 2020 IEEE/CVF Conference on Computer Vision and Pattern Recognition (CVPR).

[27] Ye M., Xu S., Cao T. HVNet: Hybrid voxel network for LiDAR based 3D object detection. Proceedings of the IEEE/CVF Conference on Computer Vision and Pattern Recognition (CVPR).

[28] Sindagi V.A., Zhou Y., Tuzel O. (2019). Mvx-net: Multimodal voxelnet for 3d object detection. Proceedings of the 2019 International Conference on Robotics and Automation (ICRA).

[29] Vora S., Lang A.H., Helou B., Beijbom O. Pointpainting: Sequential fusion for 3d object detection. Proceedings of the IEEE/CVF Conference on Computer Vision and Pattern Recognition.

[30] Wang C., Ma C., Zhu M., Yang X. PointAugmenting: Cross-Modal Augmentation for 3D Object Detection. Proceedings of the 2021 IEEE/CVF Conference on Computer Vision and Pattern Recognition (CVPR).

[31] Chen X., Ma H., Wan J., Li B., Xia T. Multi-view 3d object detection network for autonomous driving. Proceedings of the IEEE Conference on Computer Vision and Pattern Recognition.

[32] Liang M., Yang B., Wang S., Urtasun R. Deep continuous fusion for multi-sensor 3d object detection. Proceedings of the European Conference on Computer Vision (ECCV).

[33] Liang M., Yang B., Chen Y., Hu R., Urtasun R. Multi-task multi-sensor fusion for 3d object detection. Proceedings of the IEEE/CVF Conference on Computer Vision and Pattern Recognition.

[34] Yoo J.H., Kim Y., Kim J., Choi J.W. (2020). 3d-cvf: Generating joint camera and lidar features using cross-view spatial feature fusion for 3d object detection. Computer Vision—ECCV 2020: Proceedings of the 16th European Conference, Glasgow, UK, 23–28 August 2020.

[35] Pang S., Morris D., Radha H. (2022). CLOCs: Camera-LiDAR object candidates fusion for 3D object detection. Proceedings of the 2020 IEEE/RSJ International Conference on Intelligent Robots and Systems (IROS).

[36] Selesnick I.W., Baraniuk R.G., Kingsbury N.C. (2005). The dual-tree complex wavelet transform. IEEE Signal Process. Mag..

[37] Yu W., Zhou P., Yan S., Wang X. (2023). Inceptionnext: When inception meets convnext. arXiv.

[38] Tong Z., Chen Y., Xu Z., Yu R. (2023). Wise-IoU: Bounding box regression loss with dynamic focusing mechanism. arXiv.

[39] Charron N., Phillips S., Waslander S.L. (2018). De-noising of lidar point clouds corrupted by snowfall. Proceedings of the 2018 15th Conference on Computer and Robot Vision (CRV).

[40] Pitropov M., Garcia D.E., Rebello J., Smart M., Wang C., Czarnecki K., Waslander S. (2021). Canadian adverse driving conditions dataset. Int. J. Robot. Res..

[41] Geiger A., Lenz P., Stiller C., Urtasun R. (2013). Vision meets robotics: The kitti dataset. Int. J. Robot. Res..

[42] Geiger A., Lenz P., Urtasun R. (2012). Are we ready for autonomous driving? the kitti vision benchmark suite. Proceedings of the 2012 IEEE Conference on Computer Vision and Pattern Recognition.

